# Seroprevalence and Risk Factors of Small Ruminant Brucellosis in West Hararghe Zone of Oromia Regional State, Eastern Ethiopia

**DOI:** 10.1155/2021/6671554

**Published:** 2021-03-23

**Authors:** Umer Seid Geletu, Munera Ahmednur Usmael, Yesihak Yusuf Mummed

**Affiliations:** ^1^College of Agriculture, Oda Bultum University, P.O. Box 226, Chiro, Ethiopia; ^2^Oromia Bureau Livestock and Fishery Resources, West Hararghe Zone, Chiro Wereda, P.O. Box 226, Chiro, Ethiopia; ^3^School of Animal Science and Randge Land, Haramaya University, Dire Dawa, Ethiopia

## Abstract

A cross-sectional study design was employed on collected sera samples to investigate brucellosis in small ruminants from December 2018 to November 2019 with the objectives of estimating the seroprevalence and potential risk factors for the occurrence of brucellosis in small ruminants in selected districts of West Hararghe: Chiro, Hirna, and Mieso. A total of 2070 collected sera samples from small ruminants were tested using serological tests and screened by RBPT and confirmatory test (CFT). The overall seroprevalence of the present study was 0.24% in small ruminants (Chiro 0.2%, Hirna 0%, and Mieso 0.3%). The chi-square test (Stat 14.0) was used to determine the strength of potential risk factors associated with the occurrence of brucellosis by using univariable logistic regression. Mixed flock (OR = 2.11 (1.33–3.36 CI; *P*=0.002)), agropastoral (OR = 4.01 (2.35–6.84 CI; *P*=0.0001)) and pastoral (OR = 2.59 (1.37–4.90 CI; *P*=0004)) production system, and larger flock size (OR = 1.68 (1.08–2.60 CI; *P*=0.021) were factors significantly affecting the prevalence of small ruminant brucellosis. Univariable analysis was used, and independent predictors of small ruminant brucellosis were further analyzed using multilogistic regression. This disease was presented in the current study area; therefore, the careful separation of positive animals would help to prevent and control further distribution of the disease.

## 1. Introduction

Ethiopia is one of the countries which endowed the largest livestock population in Africa. Livestock production varies due to differences in resource endowment, climatic conditions, and human and livestock population, level of economic development, research support, and government economic policies. Livestock in Ethiopia provides draught power, income for farming communities, means of saving, and investment and is an important source of foreign exchange earnings for the nation. The sector provides an estimated 16% of total GDP (equivalent to 30% of the agricultural GDP) and generates 14% of the country's foreign exchange [1].

The function and purpose for which livestock are reared vary considerably across the major agroecological and socioeconomic zones and the major livestock production system, the highland crop-mixed farming, and lowland pastoral and agropastoral production system. Usually, the pastoral and agropastoral areas are found in the lowlands and characterized by extensive production which is largely based on the rangeland [2]. The country hosts a large number of small ruminants which constitute an estimated number of 47.83 million, of which 29.12 million are sheep and 28.88 million are goats. Seventy-five percent of sheep were adapted to highlands and about 76% of goats were adapted to lowlands [3].

Small ruminants and their products are important export commodities significantly contributing to the national economy; moreover, they support the livelihood of millions of pastoral peoples as a source of milk and meat. Their adaptability to a broad range of environments, short generation cycle, and high reproductive rates that lead to the high production efficiency made small ruminant production an attractive enterprise in pastoral production system [4]. There was a growing export market for sheep and goat meat in the Middle Eastern Gulf States and African countries [5]. The main constraints to livestock development in Ethiopia are nutritional shortage, traditional husbandry, water shortage, poor marketing, and different diseases that limit productivity like brucellosis.

Brucellosis is a disease caused by infection with Gram-negative coccobacillary bacteria of genus Brucella. The disease in goats and sheep is caused by *B. melitensis*, however, *B. abortus* may cause clinical brucellosis and *B. ovis* causes epididymitis in ram. Abortion in late-term pregnancy, stillbirth, birth of weak offspring, acute orchitis, and infertility were characteristics of the disease [6].

Brucellosis is a widespread zoonosis mainly transmitted from cattle, sheep, goats, pigs, and camels through direct contact with blood, placenta fetuses, or uterine secretion or through consumption of the contaminated raw animal product (especially unpasteurized milk and soft cheeses) in endemic areas [7]. *Brucella melitensis* causes a fulminating disease in humans which is characterized by intermittent fever (undulant and Malta fever), malaise, fatigue, and osteomyelitis, is a common complication in humans, and is the most prevalent species owing in part to difficulties in immunizing free-ranging goats and sheep [8]. The distribution of different species of Brucella and their biovars varies with geographical areas. *B. abortus* is mostly widespread. *B. melitensis* and *B. suis* are irregularly distributed. *B. neotomae* was isolated from desert rat (*Neotoma lepida*) in Utah, USA, and its distribution is limited to natural foci, as the infection has never been confined in humans and domestic animals [9]. The disease is more common in countries that do not have standardized and effective public health and domestic animal health programs. Areas currently listed as high risk are the Mediterranean Basin (Portugal, Spain, Southern France, Italy, Greece, Turkey, and North Africa), South and Central America, Eastern Europe, Asia, Africa, the Caribbean, and the Middle East.

There are different factors associated with the occurrence of the disease in animals. Host factor includes age, sex, and reproductive status of the animals which can determine the susceptibility of animals to the disease. (sexually mature and pregnant animals are more susceptible) [10]. Placental trophoblast produces erythritol in increasing amount during the later stage of pregnancy which coincides with the period when pregnant cattle are more susceptible to infection. The preferential utilization of erythritol rather than glucose is a characteristic of pathogenic Brucella strains. Erythritol promotes the growth of some strains of Brucella; however, as Brucella has been found in reproductive tracts of animals with no detectable levels of erythritol, the role of this sugar in the virulence of the organism has been in question [11].

Since it is not feasible to isolate the causative organism from infected cases, serological tests, namely, the RBPT, SAT, ELISA, and CFT are important in routine diagnosis of the disease. Brucellosis, like tuberculosis, is a chronic granulomatous infection caused by intracellular organism and requires combined, protracted antibiotic treatment. The disease causes much clinical morbidity as well as considerable loss of productivity in animals in the developing world. In this era of international tourism, it becomes a common imported disease in the developed world [12]. The strategies for control and eradication of brucellosis in small ruminants were immunization to reduce the rate of infection in specified herd, elimination of infected animals by test and slaughter to obtain brucellosis-free flocks/herds and regions, prevention of spread between animals, and monitoring of brucellosis-free herds and zones [13].

Despite the presence of a large population of small ruminants in different agroecological regions of the country, limited research has been done on small ruminant brucellosis. Teshale and his colleagues [14] reported a prevalence proportion of 14.6% in sheep and 16.45% in goats in the Afar region and 1.6% in sheep and 1.7% in goats in the Somali region. Another study in the pastoral region of Afar reported a prevalence rate of 5.8% in goats and 3.25% in sheep [15]. A prevalence rate of 1.5% brucellosis in sheep was also reported from South Wollo [16] and a prevalence of 4.2% in goat was reported from South Omo [17]. A very low prevalence rate in goats (0.87%) was also reported from Bahir Dar area [18]. Generally speaking, small ruminant brucellosis in Ethiopia, particularly in West Hararghe Zone of Oromia region, was not well studied; therefore, this study was designed to know the status of small ruminant brucellosis in the study area.

### 1.1. Objectives

  (i) To determine the prevalence of small ruminant brucellosis in Chiro, Hirna, and Meiso districts of central Ethiopia
  (ii) To identify the associated potential risk factors for the occurrence of the disease in small ruminants.

## 2. Materials and Methods

### 2.1. Description of Study Areas and Population

#### 2.1.1. Study Areas

**Figure 1:**
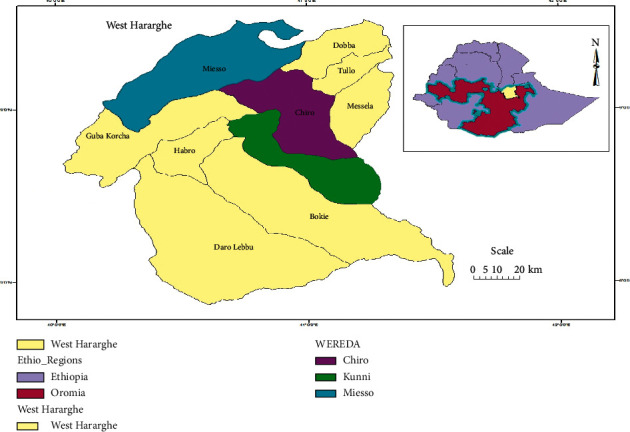
Study areas (by Umer Seid).

West Hararghe Zone is located in the eastern part of Ethiopia and Oromia 317 km far from Addis Ababa [19]. The study area is located between 7° 52′ 15″–9° 28′ 43″ N latitude and 40^0^ 03′ 33″–40° 34″ 13″ E longitude with an altitude of 1200–3600 m above sea level. It is also characterized by three agroclimatic zones, namely, highland (Dega), midland (Weina Dega), and lowland (Kola). Kola covers more percentage 49.51%, Dega covers 12.49%, and Weina Dega covers 38%. There are two rainy seasons: *ganna* (June-September) and *belgi/badhesa* (February-April). The mean annual rainfall of the area is from 650 to 1500 mm and the average temperature is from 20.5 to 24°C. The study zone has a total of 17 districts, of which 4 are pastoralist districts (Figure 1) [20].

### 2.2. Population Status and Land Use

According to the population projection of Oromia Region by Zone, released on July 1, 2015, the total human population in the study zone was estimated to be 2,467,778 (1,260,725 are men and 1,207,053 women) with an area of 17,779.4 square kilometers. In West Hararghe, 9.86% are urban inhabitants and 90.2% are rural inhabitants. A total of 395,127 households were counted in this Zone, which results in an average of 4.74 persons to a household and 380,019 housing units [20].

### 2.3. Livestock Population

Livestock are an important component of the prevailing crop-livestock mixed farming systems of the study zone. Smallholder farmers of the study area owned various livestock species such as cattle, sheep, goats, chickens, camels, and equines. The study zone has a total population of 1,017,806 cattle, 182,149 sheep, 890,226 goats, 216,819 donkeys, 1,102 mules, 1,512,784 chickens, 40,337 camels, and 65,846 beehives [21].

### 2.4. Study Design

A cross-sectional study design was employed on collected sera samples. The study was conducted from December 2018 to March 2019 with the objectives of estimating the seroprevalence and potential risk factors for the occurrence of brucellosis in sheep and goats.

### 2.5. Sampling Method and Sample Size Determination

The peasant associations (PAs) and districts were selected based on their accessibility for transportation and relative importance for small ruminant production. Farms and/or households in the PAs were selected using a random sampling strategy. All animals in the flock were sampled, if the flock had equal or less than five sheep and goats aged above six months. However, if it had more than five animals, simple random samples of 5 animals were sampled.

An expected prevalence of 2.6% for sheep and 1.83% for goats [22] and 2% absolute precision were used to calculate the required sample size followed by a two-time inflation. This is because of the absence of variance data between clusters and the interest in having a more precise estimate [23,24]. The required sample size for sheep (1101) and goat (969) was allocated to each district proportionally based on their sheep and goat population. Accordingly, 498 from Chiro, 238 from Hirna, and 1334 small ruminants from Mieso district were used to test the serum samples.

### 2.6. Sample Collection and Transportation

Blood was collected from the jugular vein of sheep and goats. Sheep and goats were aseptically bled (approximately 5 ml) from the jugular vein by using venipuncture into 10 ml vacutainer tubes which contained no anticoagulants or preservatives (BD Vacutainer Systems, Plymouth, UK). The blood samples were left for few hours at room temperature to allow clotting and then centrifuged at 3000 rpm for 10 min. The serum was collected into 1.5 ml Eppendorf tubes (Eppendorf-AG, Hamburg, Germany) and transported to National Animals Health and Investigation Center (NHADIC), using an icebox and stored at −20°C until serologically tested for the presence of anti-Brucella *antibodies*.

### 2.7. Serological Tests

#### 2.7.1. Modified Rose Bengal Plate Test (mRBPT)

All the serum samples were tested for the presence of antibodies against ovine and caprine brucellosis following the protocol of the OIE [25]. In order to improve the sensitivity of the RBPT and minimize the discrepancies between RBPT and CFT results, we used three volumes of serum and one volume of antigen (e.g., 75 *μ*l and 25 *μ*l, respectively) in place of an equal volume of each as recommended by OIE [25]. After mixing of test and control sera with the antigen, the plates/slides were rocked by hand for about 4 minutes. The results were interpreted according to Nielsen and Dunkan [26]: “0” as negative (no agglutination), “+” (barely perceptible agglutination), “++” (fine agglutination and some clearing), and “+++” (course clumping, definite with clearing).

#### 2.7.2. Complement Fixation Test (CFT)

Rose Bengal Plate Test positive sera were stored at −20°C until tested by CFT for confirmation. The protocol described in [27] which uses standard *B. abortus* antigen (Veterinary Laboratories Agency, Addlestone, United Kingdom), amboceptor (Biomerieux, France), 1% sheep RBC, and positive and negative control antisera was used. The complement was obtained from the Federal Institute for Health Protection of Consumers and Veterinary Medicine, Berlin, Germany. Sera with strong reaction at dilution of 1 : 5 with a strong reaction of approximately 100% fixation of the complement (4+), more than 75% fixation of complement (3+) at a dilution of 1 : 5, and at least 50% fixation of complement (2+) at a dilution of 1 : 10 and 1 : 20 were classified as positive [25].

### 2.8. Questionnaire Survey

A designed questionnaire (structured by both open and closed questions) was used to get associated risk factors of Brucella disease. Data on potential risk factors which include district (area), altitude, breed, sex, age, flock size, flock type (mixed flock), production system, and management system on each animal were retrospectively accessed with questionnaire format.

### 2.9. Data Management and Analysis

Data were recorded and coded in Microsoft Excel spreadsheets before transferred to statistical software for analysis (Stata^TM^ 14.0, Stata Corporation, College Station, Texas, USA). The database included serological test results and questionnaire responses. The seroprevalence of brucellosis was calculated as the number in study population testing positive to the serological test divided by the total study units tested. The chi-square (*χ*^2^) was applied to determine the existence of any association between seropositivity and potential risk factors. To measure the strength of the associations, univariable logistic regression was applied to calculate the odds ratio. All noncollinear variables from univariable logistic regression with *P* < 0.25 were further analyzed by multivariate logistic regression. For all analysis, a *P* value of <0.05 was taken as significant.

## 3. Results

### 3.1. Overall Seroprevalence

The overall seroprevalence of small ruminant brucellosis from 2070 sera samples tested was 5 (0.24%) (*χ*^2^ = 1.6526, *P*=0.438); i.e., out of 498 tested sera in Chiro district, 1 was positive by CFT (0.2%) (CI: 0.72–3.96); out of 238 tested in Hirna district, 0 was positive by CFT (0%); and out of 1334 tested in Mieso district,; 4 (0.3%) (0.61–3.03) were positive by CFT, which are shown in (Table 1).

### 3.2. Risk Factors

#### 3.2.1. Univariable Logistic Regression

Logistic regression is used to show the occurrence of the disease with its associated risk factor like district, species, age, altitude, flock size, and production system and the like.. Mieso district shows higher prevalence (0.3%) than Chiro (0.2%) and Hirna (0%) districts. Similarly, goats have a higher prevalence (0.32%) than sheep (0.18%), but this result was not statistically significant.

The prevalence of brucellosis by altitude was higher (0.3%) in lowland areas than in mid- and highlands. Females show a relatively higher prevalence of 0.25% than males; similarly, adults show a higher prevalence of 0.31% than young, which was not statistically significant. The prevalence of small ruminant brucellosis was higher in large flock size than in small flock size; similarly, agropastoral (0.31 %) and pastoral (0.3%) systems show a relatively higher prevalence than sedentary system (0%) and were statistically significant. Semi-intensive management system shows higher than 1% the extensive management system (0.1%), but not significantly significant. In other words, mixed flock has a higher prevalence of 0.37% than sheep and goats kept alone and this was statistically significant. The details of statistical output for each risk factor are summarized in Table 2.

#### 3.2.2. Multivariable Logistic Regression

The following explanatory variables were found collinear: altitude versus district, breed versus altitude, production system versus district, and altitude and mixed flock versus altitude and breed. Thus, considering collinearity, *P* < 0.25 in univariable analysis and comparable frequency of each category of every variable (>10), only production system, mixed flock, flock size, age, and species were offered to the final model. Accordingly, production system and flock size were found to be independent predictors of small ruminant brucellosis (Table 3).

## 4. Discussion

The overall seroprevalence of small ruminant brucellosis recorded in this study area is 0.24% (0.2%, 0%, and 0.3% in Chiro, Hirna, and Mieso districts, respectively). The current finding was comparable with the finding of Yeshwas et al. [16] (0.4%) in Bahir Dar, Girmay et al. [28] (0.9%) in Somali and Oromia, and Girmay et al. [28] (0.53%) in Bale and Boran. But the present finding was lower when compared with the study of Teshale et al. who reported 1.7% in goats and 1.6% in sheep in the Somali region and 14.6% in sheep and 16.45% in goats in the Afar region [28]. Since the West Hararghe was found on the border of Afra region which is fully pastoral area and was found to have higher prevalence of brucellosis in Ethiopia, these pastoralists were known with travel for searching water and pasture for their animals. It was very common to cross the West Hararghe which makes this area have a higher risk of this disease.

Serosurvey of small ruminant brucellosis shows a relatively higher prevalence in adults than in young; this is because susceptibility increases after sexual maturity especially with pregnancy, the presence of erythritol hormones and other substances in the uterus, placenta, and fetal fluids favors the proliferation of *B. melitensis* which is the principal agent causing infection in sheep and goats [6].

Small ruminants categorized in larger flock size have higher prevalence (OR = 1.68, 95% CI 1.08–2.6, *P*=0.021) than those categorized in small flock size, and this is due to close contact between animals, which contributes to the contagious nature of the infecting agent getting access to affect large number.

Mixed flock (sheep and goats kept together) shows a higher seroprevalence (OR = 2.11, 95% CI: 1.33–3.36, *P*=0.002) than sheep and goat kept alone, and this finding can agree with the following statements: as with bovine brucellosis, higher prevalence brucellosis was associated with larger, more freely mixing goat and sheep flocks in arid and semiarid pastoral areas while smaller, more restricted grazing flocks show a lower prevalence [29].

In the species category, goats show a higher seroprevalence of 0.32% (OR = 1.37, 95% CI; 0.89–2.11, *P*=0.153) than sheep (0.18%) although statistically not significant, and this is not in agreement with findings of Ashenafi et al. who reported the prevalence rate of 5.8% in goats and 3.2% in sheep in Afar region [15]. Seroprevalence of small ruminant brucellosis was significantly higher (OR = 4.15, CI: 2.23–7.72, *P*=0.000) in lowland than in mid- and highlands.

The seroprevalence of small ruminant brucellosis is significantly higher in agropastoral (OR = 4.45, 95% CI: 2.19–9.02; *P*=0.000 and pastoral (OR = 3.19, 95% CI: 1.42–7.20; *P*=0.005) systems as compared to the sedentary production system. This is in agreement with the findings of McDermott and Arimi [29].

## 5. Conclusion and Recommendations

Sheep and goat brucellosis is a zoonotic infection which was transmitted mainly by contact with discharges from the placenta and aborted material with important effects on public health, animal health, and production and is a widespread disease in the country causing serious economic loss. The serosurvey result in the study district reveals that brucellosis in small ruminants was present in a spreading infection and CFT test is the gold standard for the confirmation of brucellosis, although there was a relative difference in prevalence among districts. Sexually mature sheep and goats were affected more and this condition can greatly affect the individual and national economy, due to reduction in reproductive efficiency and infertility, which contribute to the great loss. Due to cross-infection between species of Brucella organism, keeping sheep and goats together in one flock can increase the occurrence of infection. The contagious nature of the infectious agent can increase the prevalence of infection in flocks with a large number of small ruminants. Serosurvey indicates that goats were affected more, and this condition contributes to a risk of zoonosis in areas where goat milk was consumed, especially in pastoral areas being a serious public health problem. Therefore, based on the above specified [30] conclusion, the following recommendations have been forwarded:

- Even though the seroprevalence in the study areas was not as such higher, strict control measures should be taken in order to limit the infection level
- Avoid improper handling and disposal of infective contaminated material in order to limit the spread of infection and risk of zoonosis
- Avoid the habit of drinking raw (unpasteurized) milk which was obtained from small ruminants, especially goat's milk
- Avoid mixing sheep and goats together to minimize the risk of infection.

## Figures and Tables

**Table 1:** The overall seroprevalence of small ruminant brucellosis in study districts.

Districts	Number tested	RBPT		CFT	
		Number positive	Percent seroprevalence (%)	Number positive	Percent seroprevalence (%)
Chiro	498	3	0.6	1	0.2
Hirna	238	2	0.8	0	0
Mieso	1334	10	0.75	4	0.3
Total	2070	15	0.72	5	0.24

		*χ* ^2^ = 4.3012, *P*=0.116		*χ* ^2^ = 1.6526, *P*=0.438	

**Table 2:** Univariable logistic regression analyses of explanatory variables of small ruminant brucellosis.

Variable	Level	No. tested	CFT positive (%)	OR	95% CI for CFT	Fisher's exact test *P* value
District	Hirna	238	0 (0)	1.0	—	—
	Mieso	1334	4 (0.3)	1.36	0.61–3.03	0.449
	Chiro	498	1 (0.2)	1.69	0.72–3.96	0.229
Species	Sheep	1101	2 (0.18)	1.0	—	—
	Goat	969	3 (0.32)	1.37	0.89–2.11	0.153
Altitude	Highland	301	0 (0)	1.0	—	—
	Midland	408	1 (0.25)	1.17	0.43–3.14	0.759
	Lowland	1361	4 (0.3)	4.15	2.23–7.72	0.0001
Sex	Male	442	1 (0.23)	1.0	—	—
	Female	1628	4 (0.25)	1.04	0.61–1.79	0.884
Age	Young (<1 year)	469	0 (0)	1.0	—	—
	Adult (>1 year)	1601	5 (0.31)	1.53	0.86–2.74	0.152
Flock size	Small	1097	2 (0.18)	1.0	—	—
	Large	973	3 (0.31)	1.68	1.08–2.60	0.021
Production system	Sedentary	404	0 (0)	1.0	—	—
	Pastoral	1331	4 (0.3)	2.59	1.37–4.90	0.004
	Agropastoral	335	1 (0.29)	4.01	2.35–6.84	0.0001
Management	Extensive	1785	2 (0.1)	1.0	—	—
	Semi-intensive	285	3 (1)	1.02	0.54–1.90	0.959
Mixed flock	No	1002	1 (0.1)	1.0	—	—
	Yes	1068	4 (0.37)	2.11	1.33–3.36	0.002

**Table 3:** Multivariable logistic regression analyses of potential risk factors of small ruminant brucellosis.

Variable	Level	OR (95% CI)	*P*
Species	Sheep	1.0	—
	Goats	1.17 (0.75, 1.82)	0.487
Age	Young	1.0	—
	Adult	1.43 (0.79, 2.57)	0.232
Flock size	Small	1.0	—
	Large	1.58 (1.01, 2.47)	0.048
Production system	Sedentary	1.0	—
	Pastoral	3.19 (1.42, 7.20)	0.005
	Agropastoral	4.45 (2.19, 9.002)	0.0001
Mixed flock	No	1.0	—
	Yes	1.26 (0.84, 2.48)	0.474

## Data Availability

The data used to support the findings of this study are available from the corresponding author upon request.
